# Dissemination strategies of clinical practice guidelines—mixed methods evidence synthesis protocol

**DOI:** 10.1002/gin2.70012

**Published:** 2025-02-08

**Authors:** Sumanth Kumbargere Nagraj, Tandekile Lubelwana Hafver, Ameer Hohlfeld, Emmanuel Effa, Denny Mabetha, Gertrude Kunje, Yan Jiao Shen, Carlos Zaror, Suzgika Lakudzala, Talitha Mpando, Stijn van de Velde, Thomas Agoritsas, Nicolas Delvaux, Per Olav Vandvik

**Affiliations:** ^1^ MAGIC Evidence Ecosystem Foundation, Lovisenberg gata Oslo Norway; ^2^ Centre for Evidence‐Based Health Care Stellenbosch University Stellenbosch South Africa; ^3^ Internal Medicine at the College of Medical Sciences University of Calabar Calabar Nigeria; ^4^ South African Medical Research Council, Health Systems Research Unit Cape Town South Africa; ^5^ Lilongwe University Lilongwe Malawi; ^6^ Health Management Center, General Practice Medical Center, Medical Device Regulatory Research and Evaluation Center, Chinese Evidence‐Based Medicine Center, West China Hospital Sichuan University Chengdu China; ^7^ Center for Research in Epidemiology, Economics and Oral Public Health (CIEESPO), Faculty of Dentistry Universidad de La Frontera Temuco Chile; ^8^ Kamuzu University of Health Sciences Blantyre Malawi; ^9^ Katholieke Universiteit Leuven Leuven Belgium

**Keywords:** access, barrier, channel, clinical practice guidelines, dissemination, facilitator, product

## Abstract

Clinical practice guidelines (CPGs) are shared through various dissemination strategies using a range of dissemination products and channels. However, users may have different needs for accessing and understanding them. Patients and carers from low‐ and middle‐income countries might face challenges in accessing CPGs such as inadequate systems for printed book distribution and insufficient and substandard photocopies. Many organizations offer lengthy documents, but busy healthcare workers may prefer shorter, digital versions. Digital CPGs can be sent through different channels such as email, newsletters, or social media. How users feel about these products (e.g., clinical protocol, educational material or decision aids) and how it affects the usage of CPGs is not well understood. In addition to these issues, most of the previous systematic reviews on this topic have clubbed the dissemination strategies along with the adoption of recommendations or implementation aspects. There is a need for evidence on the existing dissemination strategies disentangled from the implementation aspects. We aim to conduct a mixed‐methods systematic review to identify documented dissemination strategies for CPGs, barriers and facilitators to access such strategies and the expectations and needs of end users regarding dissemination needs. We will search literature from MEDLINE, Embase, CINAHL, Web of Science, Scopus, Epistemonikos, Agency for Healthcare Research and Quality and Medical Guidelines Clearing house. We will critically appraise all the included studies using appropriate tools based on the study design. We will use manifest content analysis to identify documented dissemination strategies and latent content analysis to understand the barriers, facilitators, preferences of end‐users. We intend to follow the convergent matrix model approach for this mixed methods evidence synthesis. We anticipate that this mixed‐methods systematic review will highlight the various strategies of dissemination of CPGs and the associated barriers and facilitators.

## Highlights

- Different stakeholders have different needs for accessing clinical practice guidelines and have different levels of understanding. In view of this, suitable types of dissemination channels and dissemination products needs to be identified for all groups of stakeholders.
- How these stakeholders feel about the products such as clinical protocol, educational material or decision aids and how it affects the usage of clinical practice guidelines is not well understood.
- Majority of the previous systematic reviews on this topic have clubbed the dissemination strategies along with the adoption of recommendations or implementation aspects. There is a need for evidence on the existing dissemination strategies disentangled from the implementation aspects.
- This systematic review protocol explains the methods related such a mixed‐methods evidence synthesis.

## 1 BACKGROUND

According to the United States of American Institute of Medicine, clinical guidelines are “statements that include recommendations intended to optimize patient care that is informed by a systematic review of evidence and an assessment of the benefits and harms of alternative care options”.^1^ Clinical practice guidelines (CPGs) can help make healthcare more consistent and safer when developed and used correctly. They offer best practices for treating patients, set standards for health professionals, aid in training, and empower patients to make informed choices.^2^

Transfer of such evidence‐based recommendations in CPGs into practice is always slow.^3^ Nevertheless, a swift uptake of best practices for treating patients is possible only if healthcare professionals are aware of the existence of such CPGs and are able to access and comprehend the recommendations. Therefore, during the planning phase of CPGs, the Scottish Intercollegiate Guidelines Network (SIGN) advises to devise dissemination plans in parallel with the development of CPGs.^4^

Dissemination is defined as “the intent is to spread knowledge and the associated evidence‐based interventions to enhance the adoption and the implementation of the information and/or intervention“.^5^, ^6^ Disseminating CPGs is an important step to implementing evidence‐based innovations in clinical care. This definition implied that the adoption and implementation are the sequence of the dissemination. However, it did not clearly differentiate dissemination from adoption and implementation, and ‘dissemination’ and ‘implementation’ have been used interchangeably.^7^ In view of this, Vernooij and colleagues further redefined dissemination of health‐related information as “the active, tailored, and targeted distribution of information or interventions via determined channels using planned strategies to a specific public health or clinical practice audience, and has been characterized as a necessary but not sufficient antecedent of knowledge adoption and implementation”. ^8^

Dissemination includes a range of different strategies such as preparing products related to CPGs and disseminating these products through appropriate channels.^9^, ^10^

**Figure 1:**
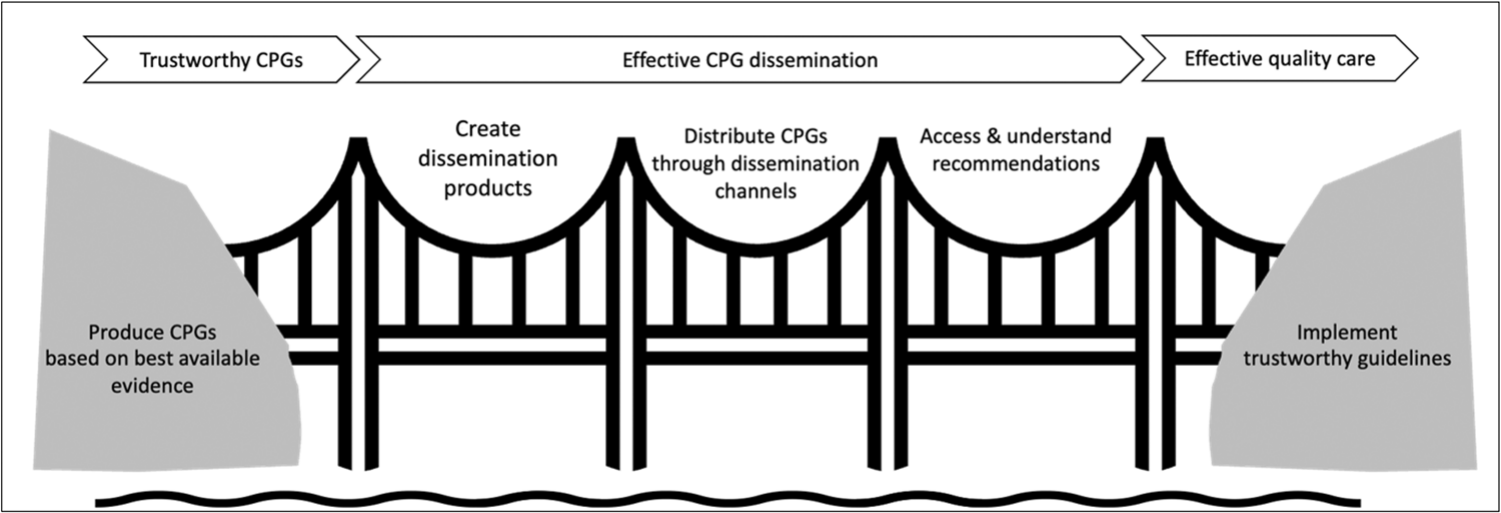
Effective CPG dissemination is a bridge between production of evidence‐informed guidelines and implementation of effective quality healthcare.

Effective CPG dissemination requires that the dissemination products are accessible and usable for the targeted audience. Figure 1 shows how effective dissemination is the bridge between trustworthy CPGs and effective quality care. It involves the creation of dissemination products and the ability of the target audience to access and understand the information and adhere to the guidance. If dissemination strategies are well planned, it can increase the access and subsequently adherence to the CPGs and thus improve the health‐related outcomes.^11^, ^12^, ^13^, ^14^ Nevertheless, an effective dissemination is neither a guarantee for adoption of CPGs nor a prediction of implementation of the recommendations. Barriers to adherence to CPGs that are related to dissemination strategies include language barriers and time constraints to access CPGs.^15^

Additional obstacles include suboptimal dissemination of printed materials, inadequate and substandard photocopying practices, linguistic incongruities such as the presence of complex language and absence of summarizations, unavailability of materials in local languages, unsupportive auditing protocols, restricted engagement of end‐users in guideline formulation, and sporadic training initiatives with uncertain dissemination to all relevant providers.^16^ To overcome some of these barriers, guideline developers can improve the dissemination of CPGs by optimal designing of the CPG products and using suitable channels.

Many organisations present their CPGs as documents which are often lengthy. It is clear that such products are not always well received by end users.^17^, ^18^, ^19^ Increasingly, CPGs are digitally developed and structured, allowing for various dissemination products.^20^ Because of such a demand for digital CPGs, The Centers for Disease Control and Prevention, USA (CDC) has initiated *“*Adapting Clinical Guidelines for the Digital Age” initiative.^21^ Nevertheless, a systematic review showed lack of evidence on the effective strategies for CPG dissemination.^22^

Despite the large variety of dissemination strategies across the world, there is a lack of comparative research evidence to inform communication and dissemination of evidence, including uncertain evidence.^23^ Also, there is a need to disentangle the evidence related to the dissemination strategies from the evidence related to adoption or implementation of CPGs.

To our knowledge, there is no comprehensive systematic review on how the various dissemination strategies are received and perceived by end users or how they impact the uptake. Therefore, we planned this mixed‐methods systematic review which will investigate dissemination strategies, barriers and enablers of these dissemination strategies and the end‐user preferences of dissemination strategies.

## 2 OBJECTIVES

This systematic review will explore the dissemination strategies of clinical practice guidelines (CPGs) in relation to end users (Table 1). Specifically, the objectives of this systematic review are:

(a) To identify how CPGs are currently disseminated to end users.
(b) To identify how CPGs are accessed and used by end users.
(c) To identify barriers and facilitators of effective CPG dissemination to end users.
(d) To identify preferences and unmet needs of end users with respect to dissemination of CPGs.

**Table 1:** Describing the SPIDER components.

SPIDER component	Outline of the component in this review
S = Sample	S = End users (Patients and the public, healthcare providers, purchasers such as employers, payers such as insurers, policy makers, product makers of drugs and devices and principal investigators^24^)
P I = Phenomenon of interest	P I = Dissemination mechanisms (products, and channels) (Operational definitions)
D = Design	D = survey, interview, focus group, case study, quantitative studies, qualitative studies, systematic reviews, policy documents
E = Evaluation	E = current dissemination mechanisms, end users current ways of accessing, barriers and facilitators for effective dissemination and end‐user preferences and expectations
R = Research type	R = Quantitative, qualitative and mixed methods studies

Depending on the availability of data, the systematic review will analyse the specific objectives in different geographical, cultural, and socioeconomic settings.

## 3 METHODS

### 3.1 Criteria for considering studies for this review

We will use the SPIDER (Sample (participants), Phenomenon of Interest (interventions), Design (types of studies), Evaluation (outcomes), Research type) framework to design the search strategy for this systematic review^25^ because of its suitability for qualitative and mixed‐method studies where the research question concern attitudes and experiences rather than quantitative outcomes. The following table (Table 1) will outline the SPIDER components in this review:

Inclusion criteria:

– Original research published from the year 1996 (as the first documented online guidelines^26^).
– Case studies, surveys, quantitative studies (RCTs, experimental studies), qualitative studies, mixed‐methods studies, systematic reviews and policy documents.
– Studies related to dissemination strategies targeting end users.
– Studies including CPG products available to organisations and/or guideline developers.
– Studies published in foreign language if full text is available as digital copies readable by translation software (scan copies of articles are not readable by translation software).
  Exclusion criteria
– Scan copies of foreign language articles.
– Narrative reviews, editorials, and commentaries.
– Scholarship activities such as CPGs disseminated in conferences or seminars.

### 3.2 Sample (Types of participants)

We will include end users as described in Table 1.

### 3.3 Phenomena of interest

Dissemination strategies (products, and channels, see Operational definitions).

### 3.4 Design (Types of studies)

We will include surveys, interviews, focus groups, case studies, quantitative studies, qualitative studies, systematic reviews and policy documents.

### 3.5 Evaluation (Types of outcome measures)

We have described the types of outcome measures for each of the review objectives in the following table (Table 2).

**Table 2:** Listing the objectives and outcome measures.

Objectives	Outcome measures
To identify how CPGs are currently disseminated to end users.	Type of dissemination products. Type of dissemination channels.
To identify how CPGs are accessed and used by end users.	Acquisition method (i.e., the channels and products end users use to get CPGs). Utilization (i.e., extent and frequency of access).
To identify barriers and facilitators of effective CPG dissemination to end users.	Type of barrier (i.e., an aspect of a dissemination mechanism that hinder access, understanding and use by end users). Type of facilitators (i.e., aspects of a dissemination mechanism that improve the access, understanding and use by end users).
To identify preferences and unmet needs of end users with respect to dissemination of CPGs.	Utility (i.e., the satisfaction of end users with dissemination mechanisms with regard to understanding and meeting information needs). Usefulness (i.e., the end users' perceptions on dissemination mechanisms' benefits and effect on care). Unmet needs related to dissemination products, for each type of end‐user. Unmet needs related to dissemination channels, for each type of end‐user.

### 3.6 Types of settings

We will include any setting where CPGs are disseminated to healthcare providers or patients/caregivers, parents, policy and decision makers, product makers and funders.

### 3.7 Search methods for identification of studies

We designed the search strategy similar to the strategy described by.^27^ We will search the databases MEDLINE via PubMed, Embase, Web of Science (Core Collection, SciELO), Scopus and CINAHL.

In addition to these, we will also search grey literature in:

- AHRQ (https://www.ahrq.gov/prevention/guidelines/index.html).
- Medical guideline clearing house (https://www.guidelinecentral.com/guidelines/).

For systematic reviews we will search in Epistemonikos. Our search strategy (Appendices) was peer‐reviewed and modified by Information specialists from KU Leuven University, Belgium.

### 3.8 Protocol registration

We have registered the protocol^28^ in PROSPERO (https://www.crd.york.ac.uk/prospero/) which is an International prospective register for systematic reviews (CRD42023470012).

### 3.9 Study selection

Five pairs of review authors will independently screen the titles and abstracts of identified studies for eligibility. The full text of all potentially eligible studies will be retrieved and screened independently by four pairs of review authors. Any disagreements will be resolved by discussion or through consultations with the third review author who will act as an arbiter (P.O.V. or N.D). A PRISMA (Preferred Reporting Items for Systematic reviews and meta‐analyses) flowchart will be used to show the studies identified, screened, and included in qualitative synthesis.

If there are multiple reports of a single study, we will combine them and consider as one study. This will be expressed number of reports and number of studies in the PRISMA flow chart.

If we end up with more than 50 included studies, we will consider using a stratified purposive sampling approach as part of the qualitative research part.^29^, ^30^ We will stratify the list of included studies based on suitability of title and abstracts for each of the objectives.

### 3.10 Data extraction

Three pairs of authors will independently extract the data from the included studies in a customised data extraction sheet. Any disagreements between these two authors will be resolved through discussion. If the authors are unable to resolve their disagreements, then a third reviewer will act as an arbiter.

We will not exclude any studies that satisfy our inclusion criteria based on the reported outcomes of interest.

We will contact the corresponding authors via e‐mail if relevant data is missing. If we do not hear from them after a reminder mail, we will mention the respective data as missing in the data extraction sheet. We will not contact the corresponding authors if there is no e‐mail address mentioned.

We will model our data extraction on a series of potentially included papers to clearly define the various concepts relevant to our review questions on dissemination strategies (Operational definitions).

### 3.11 Critical appraisal

We will use the Critical Appraisal Skills Programme (CASP) Qualitative Studies Checklist to assess the quality of included studies.^31^ We intend to use relevant quality assessment tools based on the type of quantitative studies (such as risk of bias instrument for cross‐sectional surveys of attitudes and practices,^32^ AMSTAR‐2 (Assessing the Methodological quality of Systematic Reviews – 2) for systematic reviews^33^ and mixed method appraisal tool^34^). Three pairs of authors will appraise the quality of included studies in duplicate. Any disagreements will be resolved by discussion or through consultations with the third review author who will act as an arbiter (P.O.V or N.D).

We understand that the grey literature may not undergo the same level of peer review and quality control as academic publications and each organisation producing grey literature would follow varying structure. We are also aware that grey literature such as unpublished randomised controlled trials are appraised using ACCODS checklist.^35^ This checklist is not suitable to appraise the policy documents which we plan to include in this systematic review. In view of this, we decided not to critically appraise the grey literature included in this systematic review.

### 3.12 Certainty of evidence

We will be using GRADE CERqual^36^ for the objectives ‘c’ and ‘d’ (Barriers and facilitators and Preferences and expectations of end users) to assess the certainty of evidence. We will present summary of findings table for all these objectives.

### 3.13 Data analysis and triangulation

We will use the manifest content analysis method^37^ to map the first two objectives. We will identify different dissemination channels and products, acquisition methods, utilisation and use of CPGs from the included studies. We will identify these keywords. For the latter two objectives, we will use the latent content analysis method.^38^, ^39^ From the randomised controlled trials, we will present the effectiveness data for all the available comparisons as a table. We will interpret the findings from the included studies to compile the various barriers and facilitators for effective CPG dissemination to end‐users and their preferences and needs. We will present the results for these two objectives as ‘themes’ and explain the findings for each of the themes.

We will use the convergent integrated approach for the triangulation of quantitative and qualitative data. We will create a convergence‐coding matrix to cross‐tabulate the dissemination strategies and the identified barriers, facilitators, end‐user preferences and needs.

Based on the availability of the data, we will stratify the results based on the types of end users. Depending on the identification of relevant data from quantitative studies, we will perform subgroup analysis for different geographical, cultural, and socioeconomic settings.

### 3.14 Publication bias

We do not anticipate any publication bias in this systematic review unlike clinical trials.

### 3.15 Sensitivity analysis

We will not be performing sensitivity analysis in this systematic review.

## 4 RESULTS

We will present results of the search as appendices. We will present the results of the screening in the PRISMA flow chart. We will present the details of the included studies and the quality appraisal as tables. We will present results of the first two objectives as a comprehensive list. The results of latter two objectives will be presented qualitatively as themes.

## 5 DISCUSSION

Various guideline producing bodies have published CPGs for the past three decades and have been using a variety of dissemination strategies. Access to CPGs has evolved a lot since the advent of guideline development, however, many dissemination strategies still rely heavily on classic mechanisms such as journal publications or presentations. It is unclear how more novel mechanisms that involve social media, video, multi‐layered format, etc can influence dissemination. There remains a lack of evidence on available dissemination strategies and what barriers or facilitators influence them.

This systematic review will use a comprehensive search strategy, including multiple study designs and grey literature and using mixed‐methods approach to triangulate both quantitative and qualitative data. In addition to these, we aim to use appropriate critical appraisal tool for each of the included study designs.

The findings of this mixed‐methods review will act as a reference to understand dissemination strategies. This, in turn, will assist institutions or organisations tasked with developing or implementing CPGs, in selecting and developing appropriate dissemination strategies. A deeper understanding of dissemination strategies will facilitate the alignment of the needs of end users with the efforts of CPG developers and implementers.

## AUTHOR CONTRIBUTIONS

**Sumanth Kumbargere Nagraj:** Conceptualisation; data curation; formal analysis; project administration and writing—original draft. **Tandekile Lubelwana Hafver:** Conceptualisation; data curation; writing—original draft; writing—review and editing. **Ameer Hohlfeld:** Data curation; resources; data curation; writing—reviewing and editing. **Emmanuel Effa:** Data curation; resources; data curation; writing—reviewing and editing. **Denny Mabetha:** Data curation; resources; data curation; writing‐reviewing and editing. **Gertrude Kunje:** Data curation; resources; data curation; writing—reviewing and editing. **Yan Jiao Shen:** Data curation; resources; data curation; writing‐reviewing and editing. **Carlos Zaror:** Data curation; resources; data curation; writing—reviewing and editing. **Suzgika Lakudzala:** Data curation; resources; data curation; writing—reviewing and editing. **Talitha Mpando:** Data curation; resources; data curation; writing—reviewing and editing. **Stijn van de Velde:** Conceptualisation; data curation; resources; data curation; writing—reviewing and editing. **Thomas Agoritsas:** Conceptualisation; writing—reviewing and editing. **Nicolas Delvaux:** Conceptualisation; project administration; writing—reviewing and editing; supervision and visualisation. **Per Olav Vandvik:** Conceptualisation; funding acquisition and project administration.

## OPERATIONAL DEFINITIONS

- Dissemination product: The Cochrane collaboration defines a Cochrane dissemination product “as any piece of communication that aims to present the findings of a Cochrane Review to any target audience with the aim of supporting an informed decision”^40^ and list them as review summaries, podcasts, press releases and social media posts. In the context of this systematic review, we have modified the definition by Cochrane collaboration as journal articles, infographics, flowcharts, summaries, newsletters, clinical protocols, presentations, webinars, brochures, educational material and decision aids. This also includes aggregate products (such as patient leaflets, posters, knowledge clips, etc) that may not contain the recognizable recommendations, but rather information derived from these recommendations.
- Dissemination channel: Brownson's model for dissemination of research identifies four attributes to a dissemination process: a source, an audience, a message and a channel.^9^ The dissemination channel defines the method used to reach the audience and can include a series of possibilities, such as mass media, personal contacts, information service systems, training and educational programs. The channel represents the dimension of connectivity in Milton's classification and includes ‘collect’ approaches in which knowledge is recorded and passively diffused or pulled by the audience versus ‘connect’ approaches where knowledge is communicated more directly or pushed.^41^
- Manifest content analysis: It involves examining tangible data that is directly observable and quantifiable without interpreting any underlying meaning. This method is particularly useful in scenarios where researchers seek to document and analyse the visible, straightforward elements of texts, images, or any other communicative media.^42^

## COLLABORATORS

- Professor Tamara Kredo. South African Medical Research Council, South Africa
- Dr Amanda Brand. Senior Lecturer, Centre for Evidence‐Based Health Care, Stellenbosch University, South Africa

## CONFLICT OF INTEREST STATEMENT

MAGIC Evidence Ecosystem Foundation is a participant in the GELA project under which this review is planned. MAGICapp is used in this project to author, publish and disseminate the recommendations which are of key interest to MAGIC.

## ETHICS STATEMENT

Not applicable.

## Supporting information

Supporting information.

Supporting information.

## Data Availability

The data that support the findings of this study are openly available in PROSPERO at https://www.crd.york.ac.uk/PROSPEROFILES/470012_STRATEGY_20231114.pdf, reference number CRD42023470012.
