# Supplementary material for: Dissemination strategies of clinical practice guidelines—mixed methods evidence synthesis protocol
Source: Clin Public Health Guidel. Author manuscript; Available in PMC 2025 Mar 11. (PMC7617474; doi:10.1002/gin2.70012)
Supplement: Supporting Information [file EMS203438-supplement-Supporting_Information.zip › gin270012-sup-0001-dissemination_sr_compiled_search_strategies_20231108.pdf]

Appendix 1: Search strategy for PubMed (including Medline)
#1: ("Practice Guidelines as Topic"[Mesh] OR "guideline*"[tiab])
#2: ("Information Dissemination"[Mesh] OR (“dissemination strategy"[tiab:~6] OR “dissemination strategies"[tiab:~6]))
#3: #1 AND #2
#4: ("guideline disseminate"[tiab:~6] OR "guideline disseminating"[tiab:~6] OR "guideline disseminated"[tiab:~6] OR "guideline dissemination"[tiab:~6] OR "guidelines disseminate"[tiab:~6] OR "guidelines disseminating"[tiab:~6] OR "guidelines disseminated"[tiab:~6] OR "guidelines dissemination"[tiab:~6])
#5: ("guideline distribute"[tiab:~6] OR "guideline distributed"[tiab:~6] OR "guideline distributing"[tiab:~6] OR "guideline distribution"[tiab:~6] OR "guidelines distribute"[tiab:~6] OR "guidelines distributed"[tiab:~6] OR "guidelines distributing"[tiab:~6] OR "guidelines distribution"[tiab:~6])
#6: ("guideline uptake"[tiab:~6] OR "guidelines uptake"[tiab:~6])
#7: (#3 OR #4 OR #5 OR #6)
#8: #7 AND 1996:2030[pdat]

Appendix 2: Search strategy for Embase (Embase.com)
#1: (('practice guideline'/de) OR ('guideline*':ti,ab,kw))
#2: (('information dissemination'/de) OR (('disseminat*') NEAR/7 ('strateg*')):ti,ab,kw)
#3: (#1 AND #2)
#4: (('guideline*') NEAR/7 ('disseminat*' OR 'distribut*' OR 'uptake')):ti,ab,kw
#5: (#3 OR #4)
#6: #5 AND [1996-2023]/py
#7: #6 NOT 'conference abstract':it

Appendix 3: WoS Core Collection (Science citation index expanded - 1955 to present; Social Sciences Citation Index - 1956 to present; Arts and Humanities Citation Index – 1975 to present; Conference Proceedings Citating Index – Science – 1990 to present; Conference Proceedings Citating Index – Social Science and Humanities – 1990 to present; Emerging Sources Citating Index – 2018 to present)

#1: TS=("guideline*")
#2: TS=(("disseminat*") NEAR/7 ("strateg*"))
#3: (#1 AND #2)
#4: TS=(("guideline*") NEAR/7 ("disseminat*" OR "distribut*" OR "uptake"))
#5: (#3 OR #4)
#6: #5 AND PY=(1996-2023)
#7: #6 NOT DT=("meeting abstract")

Appendix 4: Scopus

#1: TITLE-ABS("guideline*") OR AUTHKEY("guideline*")

#2: TITLE-ABS(("disseminat*") W/7 ("strateg*")) OR AUTHKEY(("disseminat*") W/7 ("strateg*"))
#3: (#1 AND #2)
#4: TITLE-ABS(("guideline*") W/7 ("disseminat*" OR "distribut*" OR "uptake")) OR AUTHKEY(("guideline*") W/7 ("disseminat*" OR "distribut*" OR "uptake"))
#5: (#3 OR #4)
#6: #5 AND PUBYEAR AFT 1995

Appendix 5: CINAHL (via EBSCOhost)

S1: (MH "Practice Guidelines") OR TI("guideline*") OR AB("guideline*")
S2: (MH "Selective Dissemination of Information") OR TI(("disseminat*") N7 ("strateg*")) OR AB(("disseminat*") N7 ("strateg*"))
S3: (S1 AND S2)
S4: TI(("guideline*") N7 (“disseminat*” OR “distribut*” OR “uptake”)) OR AB(("guideline*") N7 (“disseminat*” OR “distribut*” OR “uptake”))
S5: (S3 OR S4)
S6: S5 AND PY 1996-2023

Expanders - Apply equivalent subjects

Appendix 6: WoS SciELO citation index (2002-present)

#1: TS=("guideline*")
#2: TS=(("disseminat*") NEAR/7 ("strateg*"))
#3: (#1 AND #2)
#4: TS=(("guideline*") NEAR/7 ("disseminat*" OR "distribut*" OR "uptake"))
#5: (#3 OR #4)

Appendix 7: Epistemonikos

(title:((disseminat* OR distribut* OR uptake) AND (guideline*)) OR abstract:((disseminat* OR distribut* OR uptake) AND (guideline*)))

Filters:
Publication year: 1996 to 2024
Publication type: systematic review
Pubmed Central (PMC): No PMC
